# Perceived academic anxiety and procrastination among emergency nursing students: the mediating role of cognitive emotion regulation

**DOI:** 10.1186/s12912-024-02302-3

**Published:** 2024-09-19

**Authors:** Amina Hemida Salem Ghattas, Ayman Mohamed El-Ashry

**Affiliations:** 1 Assistant professor of Critical Care and Emergency Nursing, Faculty of Nursing, Alexandria University, Alexandria, Egypt; 2 Lecturer of Psychiatry and Mental Health Nursing, Faculty of Nursing, University of Alexandria, Alexandria, Egypt

**Keywords:** Academic anxiety, Procrastination, Emergency nursing students, Mediating, Cognitive emotion regulation

## Abstract

**Aim:**

Explore the mediating role of cognitive emotion regulation strategies used by nursing students between academic anxiety and procrastination. **Design**: A descriptive correlational design was used. **Setting**: This study was carried out in the faculty of nursing at the University of Alexandria. **Sample**: The participants in this study were all students enrolled in emergency nursing during the second semester of the academic year 2021–2022 and a convenience sampling of 654 nursing students. The Sobel test was used as a statistical method to determine the significance of a mediation effect by assessing whether the relationship between perceived academic anxiety and procrastination was significantly reduced when cognitive emotion regulation was included, using coefficients and standard errors from regression models to calculate the test statistic. **Tools**: Academic Anxiety Scale, Academic Procrastination Scale, and Cognitive Emotion Regulation Questionnaire were used to collect data.

**Results:**

The study found that 46.5% of students reported high anxiety levels, with 40.1% perceiving moderate anxiety and only 11.3% experiencing low anxiety. Moreover, 80.4% of nursing students showed moderate academic procrastination. There is a positive correlation between procrastination and academic anxiety, with Adaptive and maladaptive coping strategies mediating this relationship, according to the Sobel test.

**Conclusion:**

Based on the results, it can be concluded that there is a complex causal and effect relationship between academic anxiety and procrastination. Nursing students may resort to academic procrastination as a means of coping. Therefore, reducing anxiety, correcting maladaptive behaviors, and enhancing adaptive cognitive and emotional regulation strategies could effectively reduce academic procrastination.

## Introduction

The global prevalence rates of academic anxiety in nursing students are estimated to range from approximately 14.3–56% [1]. While a certain level of anxiety can positively influence students’ performance and serve as a driving force for further accomplishments, excessive anxiety can have detrimental consequences on both academic performance and overall well-being. Academic anxiety and procrastination are commonly experienced by nursing students at all stages of their education. Academic anxiety refers to feelings of worry, tension, or dread that are linked to academic settings or tasks. These can include exams, assignments, specific subjects (like math or science), and social pressures related to schoolwork from parents or peers. Academic anxiety can show up in various ways, including physical symptoms (such as nausea, increased heart rate, and headaches), cognitive symptoms (like negative self-talk and difficulty focusing), and behavioral symptoms (such as procrastination and avoidance of academic tasks). and there is evidence indicating that it has a negative impact on academic achievement [2].

Moreover, academic procrastination is a complex phenomenon and a frequent tendency among students to delay educational activities. Students need to carry out an academic task or activity, such as writing a term paper, completing an academic project, or doing weekly assignments, but, for some reason, they fail to do an academic activity within the estimated time frame. In the context of nursing education, the occurrence of anxiety and procrastination can result in not only inadequate academic performance (low GPA) but also unfavorable emotional responses such as frustration, shame, or guilt. It is widely accepted that nursing education field is stressful and students are highly prone to experience heightened levels of anxiety, stress, and depression compared to their counterparts in other disciplines. Research studies investigating the correlation between anxiety and procrastination have indicated intricate cause-and-effect associations between these two factors [2, 3].

Specific sources of anxiety were explicitly associated with the nature of the courses, such as the emergency nursing and its content. Upon commencing their hospital clinical training in the Emergency Department (ED), students typically encounter physical manifestations of anxiety, including trembling, paleness, sweating, cold and clammy skin, emotional outbursts, fainting, and crying. These symptoms arise due to the overwhelming negative emotions of fear, insecurity, and distress that students experience, particularly first time patient care. Furthermore, additional contributing factors are categorized into academic, clinical, and personal or social factors. Academic factors encompass excessive preparation for theoretical exams and assignments that disrupt the balance between work and personal life, the disparity between theory and practice, criticism from instructors, poor performance in exams or periodic assignments, overwhelming course materials, and the need to absorb vast amounts of information within limited time frames. Moreover, the absence of proper guidance and support from instructors, deemed essential, is expected to heighten frustration and dissatisfaction among nursing students [4, 5].

Moreover, anxiety and procrastination are significantly influenced by clinical training, which holds the position of the second most influential factor. The clinical training setting, where students exhibit and enhance their clinical skills, is a multifaceted, overwhelming, and stressful environment due to its competitive and unfamiliar nature. Emergency Departments (EDs) are characterized by a substantial influx of patients, escalating service demands, overextended staff, and persistent urgency [6, 7]. While clinical training in EDs fosters competence development, it also challenges students and instructors. The intricacy of these units, combined with the immense pressure to provide care for critically ill patients, can harm students’ overall experience. Students often experience anxiety and helplessness as they navigate unfamiliar patient scenarios. Furthermore, they face heightened helplessness and nervousness when confronted with unforeseen emergencies, diseases, patient mortality exposure, and ethical dilemmas during training [8, 9].

According to various studies [10–12], several factors contribute to anxiety and procrastination among nursing students. These factors include poor time management, lack of sleep, violent behavior towards nursing students, inadequate support from instructors and peers, lack of social support due to living away from family, working in a private hospital during studies, less time for rest and recreational activities, poor eating habits, fear of failure, excessive self-imposed demands, idealism, dysfunctional defense mechanisms, perfectionist and neurotic traits. Too excessive details about contributing factors of academic anxiety compared to remaining bulk. You need a paragraph relating between anxiety and procrastination.

Students can use different cognitive strategies and personal resources to handle academic anxiety and procrastination. Cognitive emotion regulation (CER) is a term that describes the conscious ways of thinking that students use to manage their emotions in response to stressful situations. CER comprises nine strategies, five of which are adaptive and four of which are maladaptive. The choice of adaptive or maladaptive emotion regulation strategies can impact psychological and physical health and interpersonal interactions. Psychologists consider an inability to regulate emotions as the primary cause of mental and physical disorders. Researchers have identified various factors that influence the selection of emotion regulation strategies [13–15].

### Significant of the study

Cognitive emotion regulation strategies play a pivotal role in equipping nursing students with the necessary tools to effectively cope with the significant levels of stress and anxiety that are often inherent in their rigorous academic pursuits and demanding clinical placements [16]. These strategies, which encompass techniques such as positive reappraisal and cognitive restructuring, have been shown to substantially mitigate feelings of academic anxiety and procrastination, thereby enhancing the student’s emotional well-being and overall academic performance. Nevertheless, it is important to acknowledge that excessive dependence on positive reappraisal may inadvertently lead to the neglect of genuine problems that require attention, while the suppression of emotions can paradoxically result in heightened stress levels and emotional fatigue. Moreover, the utilization of non-adaptive strategies, such as rumination, has the potential to intensify stress levels, and an overemphasis on self-regulation may inadvertently diminish the capacity for empathy towards patients in clinical settings. The importance of achieving a harmonious balance between these cognitive strategies and appropriate supportive measures cannot be overstated, as it is essential for preserving mental health and delivering effective patient care [17, 18]. In light of these considerations, it becomes imperative for nursing students to be educated about the potential pitfalls of various emotion regulation strategies while simultaneously being encouraged to develop a comprehensive toolkit that promotes resilience. Fostering an environment that prioritizes emotional health and empathetic patient interactions is crucial for developing competent and compassionate nursing professionals [18].

It is unknown how the levels of perceived academic anxiety and procrastination among the emergency nursing students and coping strategies are perceived. Therefore, the study aimed to determine the prevalence of academic anxiety and procrastination and possibly determine the roles of Cognitive Emotion Regulation as a mediating coping strategy used by the students to deal with these problems.

### The present study aimed to

- Determine the levels of academic anxiety, procrastination, and Cognitive Emotion Regulation among emergency nursing students.
- Investigate the mediating role of Cognitive Emotion Regulation on the relationship between academic anxiety and academic procrastination among emergency nursing students.

### Research questions

- What are the levels of academic anxiety, procrastination, and Cognitive Emotion Regulation among emergency nursing students?
- How do Cognitive Emotion Regulation strategies mediate the relationship between academic anxiety and academic procrastination among emergency nursing students?

### Design

The current study’s data was collected using a cross-sectional correlational descriptive study design using STROBE guidelines.

### Setting

The study was conducted at Alexandria University, Egypt, Faculty of Nursing, a comprehensive institution that offers a wide range of nursing programs. The faculty operates under the authority and supervision of the Ministry of Higher Education. It provides bachelor’s, master’s, and doctorate degrees in nursing sciences, catering to the diverse needs of students. The undergraduate program courses are offered over eight semesters according to the credit hours system. The faculty includes nine specialized scientific departments: Medical-Surgical Nursing, Nursing Education, Pediatric Nursing, Critical Care and Emergency Nursing, Obstetric and Gynecological Nursing, Community Health Nursing, Gerontological Nursing, Nursing Administration, and Psychiatric Nursing and Mental Health.

### Sample

A convenience sampling of all students enrolled in the emergency nursing course (*n* = 700) during the second semester of the academic year 2022–2023 in the Faculty of nursing at Alexandria University who agreed to participate in the current study were enrolled. 46 students were refused to participate in the study.

### Instruments

#### Tool one: Socio-demographic and academic features of the students

The researcher developed this questionnaire based on a literature review to capture the basic socio-demographic information of the students. It includes age, sex, marital status, and whether you live with your family during the study. Do you work in a private hospital while studying? Do you engage in students’ faculty committees? What was your academic achievement in the first academic year?

#### Tool two: academic anxiety scale

The Academic Anxiety Scale was English scale created by Pizzie & Kraemer in 2019 to measure academic anxiety in university students [19]. The scale consists of 11 self-report questions that use a 4-point Likert scale to rate responses ranging from “Not at all” one to “Very typical.”four.

The reliability and validity of the scale have been established, with a Cronbach coefficient of 0.845. The survey identifies cut scores for different “levels” of academic anxiety based on the total score: not anxious from 11 to14, mild academic anxiety from 15 to 20, moderate academic anxiety from 21 to 29, and high academic anxiety from 30 to 40.

#### Tool three: academic procrastination scale (APS)

A measuring tool named the Academic Procrastination Scale was created in an English language and validated by McCloskey and Scielzo in 2015 [20]. It was designed to assess procrastination, specifically on academic tasks such as term papers, exams, and projects in an academic setting. The scale consists of 25 questions, each answered using a 5-point Likert scale ranging from never (1) to always (5). The APS score ranges from 25 to 125. The Academic Procrastination Scale (APS) categorizes scores into three levels. A score between 25 and 58 indicates low procrastination. Scores ranging from 59 to 91 fall into the moderate procrastination category. Finally, scores between 92 and 125 signify high procrastination, indicating a frequent tendency to delay academic responsibilities. The APS exhibits excellent reliability and internal consistency with an alpha coefficient of 0.94.

#### Tool four: cognitive emotion regulation questionnaire (CERQ)

It was an English questionnaire developed by Garnefski in 2007. It measures cognitive emotion regulation (CER) strategies individuals use to respond to life-threatening events [21]. The scale has 36 items and includes adaptive and maladaptive Cognitive Emotion Regulation strategies. The Cognitive Emotion Regulation Questionnaire (CERQ) assesses how individuals manage their emotions following negative experiences through nine subscales: self-blame, acceptance, rumination, positive refocusing, refocus on planning, positive reappraisal, putting into perspective, catastrophizing, and other-blame. Each subscale represents a different cognitive strategy, measured on a 5-point Likert scale from 1 (almost never) to 5 (almost always), with total scores ranging from 36 to 180. Scores between 36 and 84 indicate low use of cognitive strategies. Scores from 85 to 132 represent moderate use. Finally, scores between 133 and 180 signify high use. The Persian version of the CERQ scale was standardized by Hasani in 2010 for the Egyptian population. The scale’s reliability was measured using Cronbach’s alpha coefficient, and the score was between 0.51 and 0.77. Besides, exploratory factor analysis with varimax rotation and correlation between subscales (ranging from 0.32 to 0.67) reported the validity of the CERQ; its optimal criterion validity was also reported.

### Ethical considerations

The researcher obtained ethical approval from the Research Ethics Committee of the Faculty of nursing at Alexandria University (IRB00013620/12/2023) then written permission obtained from the head of the critical and emergency nursing department to be able to access for study participants. Before distributing the study surveys and collecting data. The participants were informed through voice massage that the study was voluntary, confidential, and anonymous. They had to click on the online survey link and confirm their participation agreement after reading attached instructions about study aim and how to respond on each item. The researcher was the only one who had access to the data. The study emphasized the importance of maintaining confidentiality, data safety, informed consent, and voluntary participation. Participants were assured they would face no negative consequences if they chose not to participate.

### Validity and reliability

To ensure accuracy and maintain the integrity of the study, we implemented a rigorous translation and back-translation process for the academic anxiety scale, academic procrastination scale, and cognitive emotion regulation questionnaire. Initially, a bilingual expert translated these scales from English to Arabic. Subsequently, a different bilingual expert, who was not involved in the initial translation, translated the Arabic versions back into English to identify any discrepancies. A panel of five experts, including three professors in psychiatric and mental health nursing and two experts from the critical care nursing department, reviewed the translated scales to further validate the content. They provided valuable feedback on the question structure, clarity, and validity. This feedback was meticulously analyzed and incorporated to ensure precision. Additionally, we conducted a Lawshe’s Content Validity Ratio (CVR) test to quantitatively assess the content validity of the scales, resulting in a CVR value of 0.85, indicating high content validity. The iterative process of translation, back-translation, expert review, and the CVR test helped maintain the study’s integrity and ensured that the instruments were both reliable and culturally appropriate for the target population. The Self-academic anxiety scale, academic procrastination scale, and cognitive emotion regulation questionnaire demonstrated high-reliability levels, with reliability scores of 0.83, 0.91, and 0.92, respectively.

### Pilot study

A pilot study was conducted with a subset of the sample, 10% of the students (*n* = 65). The primary objectives of this pilot study were to assess the clarity and practicality of the study tool and gauge the amount of time necessary to complete the study scale.

### Data collection

The study was done between April 2023 and June 2023. The online surveys (tools I, II, III, & IV) were created by the researcher using Google Drive Forms, The researchers obtain accessibility to all clinical Watsapp groups for communication with students. and the links were sent to all students enrolled in the accelerated program through the WhatsApp of each distinct group. The researcher included a voice message to explain the aim of each survey and was available for any explanation from students or help at the time of data collection.

### Statistical analysis

Once the data were collected, they underwent coding and were formatted for computer input. After following the data entry, rigorous checks and verifications were performed to prevent input errors. Various techniques, such as frequency analysis, cross-tabulation, and manual review, were employed to identify and rectify discrepancies. Statistical analysis was conducted using the Statistical Package for the Social Sciences (SPSS version 25), encompassing descriptive statistics such as numbers, percentages, and averages. Furthermore, regression analysis, Pearson correlation, and Sobel test were employed as statistical tests. The chosen significance level for the study was set at a *p*-value of 0.05 or less.

## Results

Table (1) shows the distribution of the studied students according to their characteristics. It was found that less than half (48.6%) of the students were less than 21 years old, while less than one-tenth (8.0%) were 23 years and older. Moreover, most (83.5%) of the students were female, and most were single and living with their families during the study (97.2% and 96.0%). Furthermore, less than one-fifth (18.7%) reported participation in extracurricular activities, and less than one-quarter (21.4%) mentioned that they work besides education. Additionally, around one-fifth (20.2%) of the students had excellent academic achievement during the last year compared to a minority (1.8%) with acceptable achievement.

**Table 1:** Distribution of the studied students according to their basic characteristics

Students’ characteristics	Total *N* = 654	
	No.	%
**Age (years)**		
■ 19- ■ 21- ■ ≥ 23	318 284 52	48.6 43.4 8.0
**Sex**		
■ Male ■ Female	108 546	16.5 83.5
**Marital status**		
■ Single ■ Married	636 18	97.2 2.8
**Living with family during the study**		
■ Yes ■ No	628 26	96.0 4.0
**Engagement in extracurricular activities**		
■ Yes ■ No	122 532	18.7 81.3
**Work beside education**		
■ Yes ■ No	140 514	21.4 78.6
**Academic achievement during the last year**		
■ Excellent ■ Very good ■ Good ■ Acceptable	132 412 98 12	20.2 63.0 15.0 1.8

Table (2) illustrates the levels and mean scores of procrastination, academic anxiety, and the use of cognitive emotion regulation strategies among the studied students. Concerning academic anxiety, a minority (2.1%) of the students had no anxiety, while less than half (46.5%) of them had a high level of anxiety, with a mean score of 28.81 ± 7.130. Regarding procrastination, less than one-fifth (14.1%) of the students had a low level, and only 5.5% had a high level, with a mean score of 70.42 ± 12.45. about the use of adaptive emotion regulation strategies, more than two-fifths (41.9%) of the students had a high level of use, while only 2.4% had a low level of use. On the other hand, it was noted that 8.9% of the students had a low level of maladaptive emotion regulation strategies compared to 13.8% of them who used them frequently.

**Table 2:** Distribution of the Studied Students according to the levels and Mean Scores of Procrastination, Academic Anxiety, and the adaptive and maladaptive Emotion Regulation Strategies

Variable	Levels			Mean ± SD
		No.	%	
Academic Anxiety	No Low Moderate High	14 74 262 304	2.1 11.3 40.1 46.5	28.81 ± 7.130
Procrastination	Low Moderate High	92 526 36	14.1 80.4 5.5	70.42 ± 12.45
Use of adaptive Emotion Regulation Strategies	Low Moderate High	16 364 274	2.4 55.7 41.9	48.73 ± 9.457
Use of maladaptive Emotion Regulation Strategies	Low Moderate High	58 506 90	8.9 77.4 13.8	48.73 ± 9.457

**Fig. 1:**
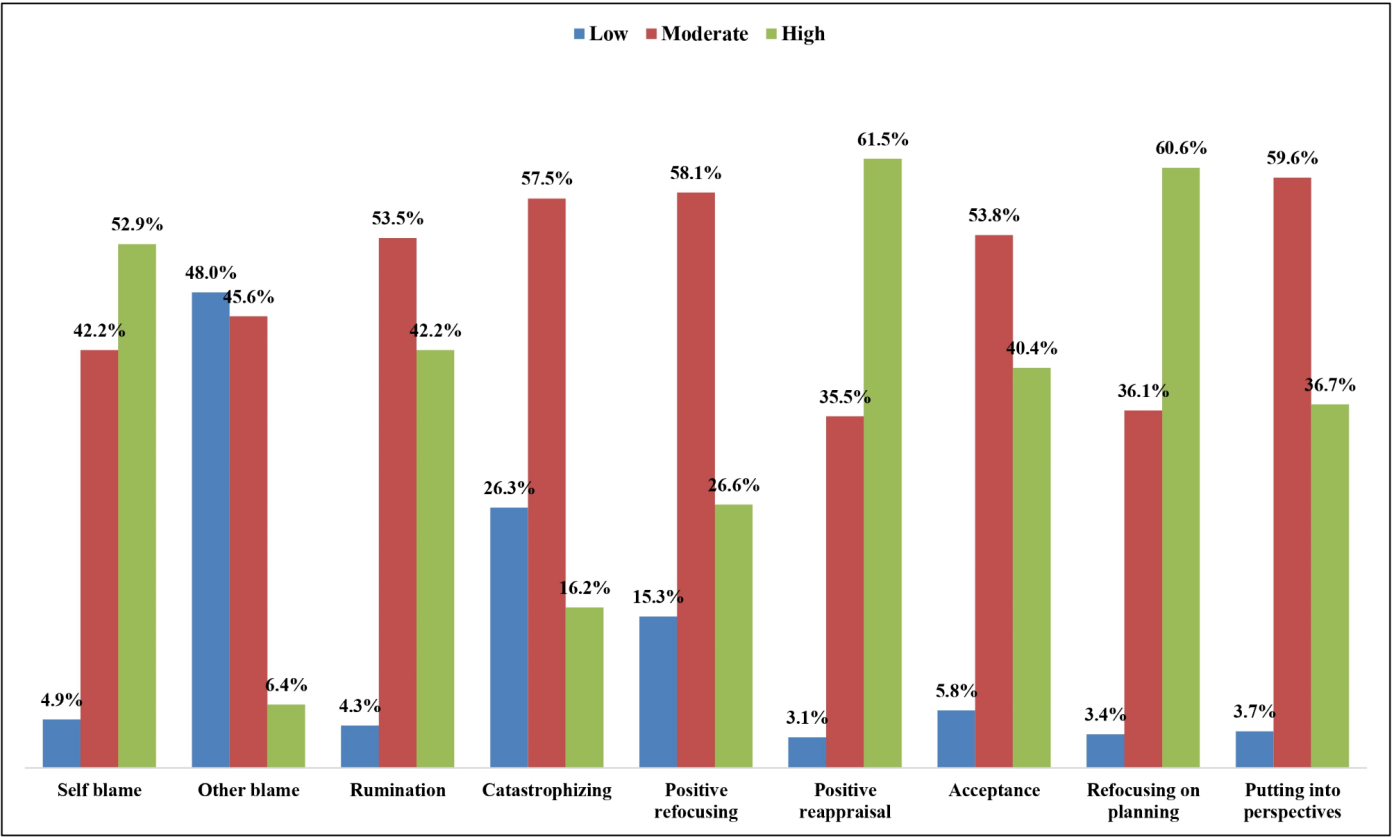
Portrays the distribution of the studied students according to the level of use of adaptive and maladaptive emotion regulation strategies

Figure (1) portrays the distribution of the studied students according to the level of use of adaptive and maladaptive emotion regulation strategies. Among the maladaptive emotion regulation strategies, it was noticed that more than half (52.9%) and more than two-fifths (42.2%) of the students had high levels of use of self-blame and rumination, respectively. While 16.2% of them were using catastrophizing frequently compared to 6.4% of them who blamed others frequently as a coping strategy for stress and anxiety. Regarding adaptive coping strategies, less than two-thirds (61.5% and 60.5%) of the students had a high level of use of positive reappraisal and refocusing on planning, respectively, while around two-fifths (40.4%) of them had a high level of use of acceptance strategy. On the other hand, 15.3% and 3.7% of the students had a low level of positive refocusing and putting into consideration strategy, respectively.

**Fig. 2:**
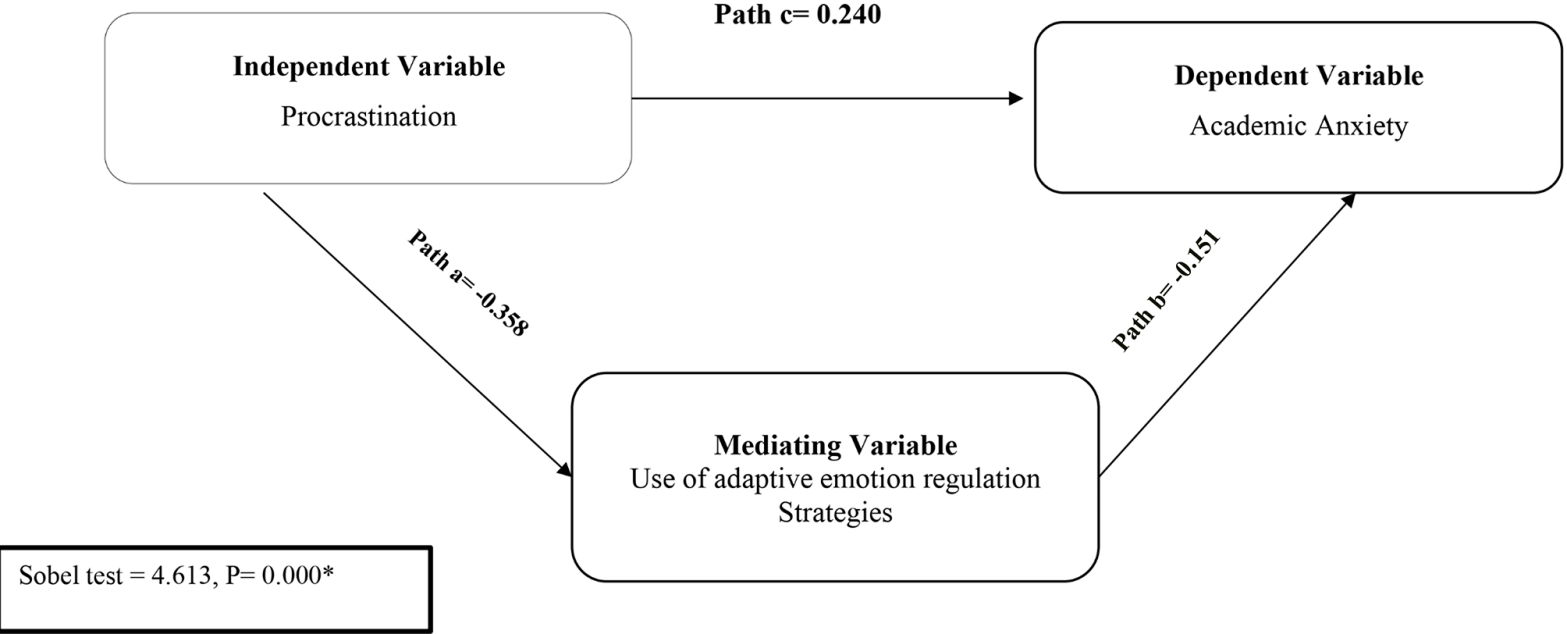
The mediating effect of students’ procrastination, academic anxiety, and the use of adaptive emotion regulation strategies. Sobel test = test of the mediating effect * Significant *p* ≤ 0.05

Tables (3, 4 &5) and Fig. (2) show the regression analysis and the mediating effect of adaptive emotion regulation strategies used between procrastination and academic anxiety. It was found that procrastination significantly predicts academic anxiety (B = 0.243, t = 11.934, *p* = 0.000). Furthermore, the use of adaptive emotion regulation strategies has a significant negative predictive effect on academic anxiety (B = − 0.151, t = 7.574, *p* = 0.000). This means that in the model, there is a significant negative correlation in the path a & b. However, the direct influence between procrastination and academic anxiety in path C is positive. Finally, the Sobel test indicates the presence of a mediating effect of the use of adaptive emotion regulation strategies between procrastination and academic anxiety (Sobel test = 4.613, *p* = 0.000).

**Table 3:** The correlation matrix between Procrastination, academic anxiety, adaptive and maladaptive emotion regulation strategies

		Procrastination	Academic Anxiety	Maladaptive coping strategies
Procrastination	*p*			
	*r*			
Academic Anxiety	p	0.423		
	r	0.000*		
Maladaptive Emotion Regulation Strategies	p	0.469	0.444	
	r	0.000*	0.000*	
Adaptive Emotion Regulation Strategies	p	-0.671	-0.265	-0.783
	r	0.017*	0.000	0.011*

r: Pearson coefficient *: Statistically significant at *p* ≤ 0.05

**Table 4:** Regression analysis between Students’ Procrastination and Academic Anxiety

Model	Unstandardized Coefficients		Standardized Coefficients	t	*P*
	B	Std. Error	Beta		
Constant	11.733	1.453		8.073	0.000*
Procrastination	0.243	0.020	0.423	11.934	0.000*
Model Summary	*R* = 0.423	R^2^ = 0.179	F = 142.431	*P* = 0.000*	
Dependent variable = Academic Anxiety		Independent variable = Procrastination			

R^2^: Coefficient of determination. t: t-test of significance. *: Statistically significant at *p* ≤ 0.05

**Table 5:** Regression Analysis between Students’ Procrastination, Adaptive Coping Skills, and Academic Anxiety

Model	Unstandardized Coefficients		Standardized Coefficients	t	*P*
	B	Std. Error	Beta		
Constant	22.615	2.002		11.296	0.000*
Procrastination	0.240	0.020	0.419	12.312	0.000*
Adaptive Emotion Regulation Strategies	-0.151	0.020	-0.258	7.574	0.000*
Model Summary	*R* = 0.496	R^2^ = 0.246	F = 106.05	*P* = 0.000*	
Dependent variable = Academic Anxiety		Independent variable = Procrastination & Use of Adaptive emotion regulation Strategies			

R^2^: Coefficient of determination. t: t-test of significance. *: Statistically significant at *p* ≤ 0.05

**Fig. 3:**
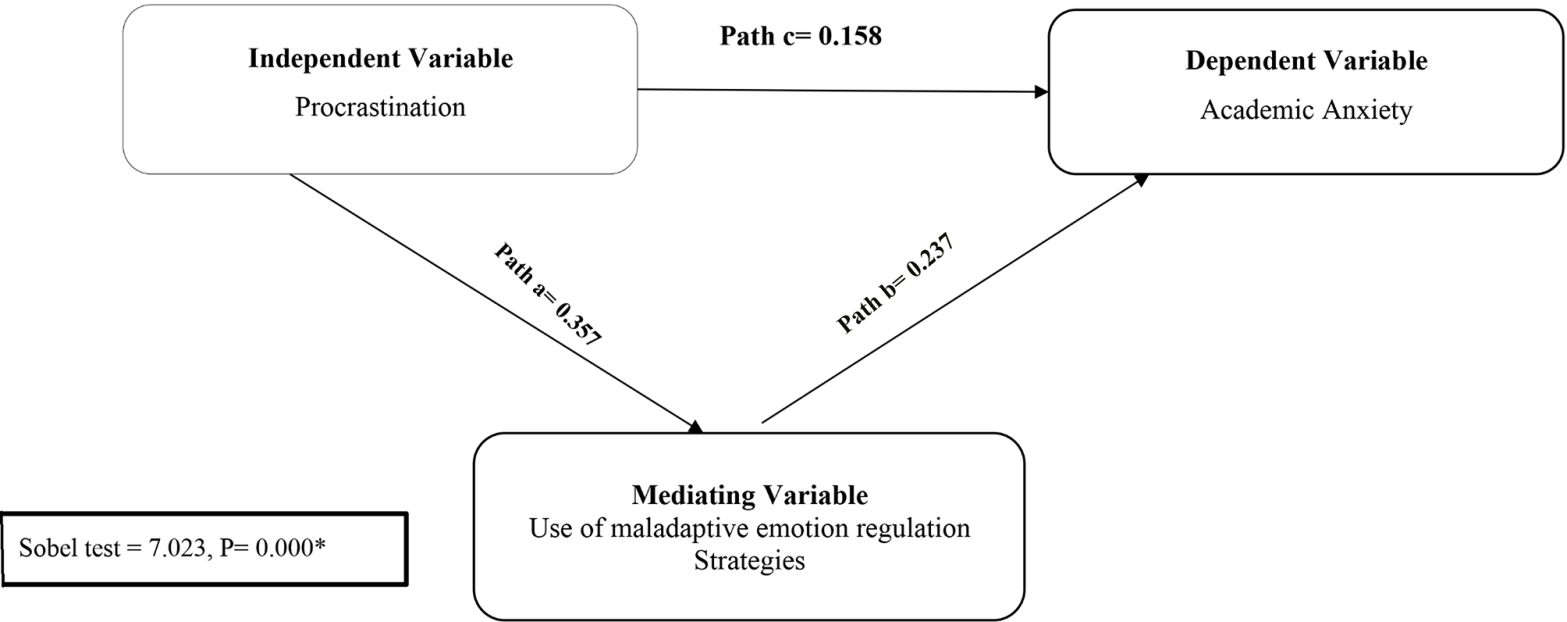
The mediating effect of students’ procrastination, academic anxiety, and the use of maladaptive emotion regulation strategies. Sobel test = test of the mediating effect * Significant *p* ≤ 0.05

Table (6) and Fig. (3) illustrate the regression analysis and mediating effect of maladaptive coping strategies used between procrastination and academic anxiety. It was noticed that procrastination had a positive effect on academic anxiety (B = 0.243, t = 11.943, *p* = 0.000). Additionally, the use of maladaptive emotion regulation strategies has a significant positive effect on academic anxiety (B = 0.237, t = 8.221, *p* = 0.000). This indicates that in the model, there is a significant positive correlation in the paths a, b, and c, which were reflected in the Sobel test as there is a mediating effect of the use of maladaptive emotion regulation strategies between procrastination and academic anxiety (Sobel test – 7.023, *p* = 0.000).

**Table 6:** Regression Analysis between Students’ Procrastination, Use of maladaptive emotion regulation Strategies and Academic Anxiety

Model	Unstandardized Coefficients		Standardized Coefficients	t	*P*
	B	Std. Error	Beta		
Constant	6.129	1.543		3.972	0.000*
Procrastination	0.158	0.022	0.276	7.205	0.000*
Maladaptive Emotion Regulation Strategies	0.237	0.029	0.315	8.221	0.000*
Model Summary	*R* = 0.506	R^2^ = 0.256	F = 112.283	*P* = 0.000*	
Dependent variable = Academic Anxiety		Independent variable = Procrastination & Use of Maladaptive emotion regulation Strategies			

R^2^: Coefficient of determination. t: t-test of significance. *: Statistically significant at *p* ≤ 0.05

## Discussion

In the emergency nursing course, students reported a high level of anxiety related to the nature and content of the course. Every day of the course, students face many stressors, such as academic workload, clinical environment-related, and personal stressors. Some nursing students may have difficulty adapting quickly due to inadequate abilities, leading to psychological burdens. Thus, when nursing students find it challenging to change this situation in the short term, they may choose a negative way instead to deal with this pressure, such as by “trying to take a break or vacation to put problems (worries) aside for a while” or by “comfort yourself.”

The current study revealed that about half of the students experienced a high level of academic anxiety, followed by more than one-third perceived a moderate level of academic anxiety. Furthermore, a vast majority of nursing students had a moderate level of academic procrastination. Additionally, the current results provided a direct relationship between academic anxiety and academic procrastination; the students with high academic anxiety had higher academic procrastination, where academic procrastination significantly predicts academic anxiety, and it has a significant negative predictive effect on the use of adaptive coping strategies.

The high level of anxiety and academic procrastination could be explained by the nature and content of the course and a lack of time management skills, lack of time to do the required assignments, to study well to keep the position in terms of grade ranking, to relax and maintain social ties, and leisure activities. Furthermore, the large amount of information to learn in short periods, too many examinations, too many assignments, worries about their level of achievement, what was expected from them, and their relation with the teaching staff and clinical instructors, “they felt scared and pressured by their instructors,” being in a very competitive and intense, pressure-filled ED environment, physical exhaustion or fatigue and spending most of their time on social media were perceived by students as reasons of anxiety and academic procrastination as well. The outcomes were delays in submitting the required assignments, academic procrastination, and feelings of guilt. The results of the current study seemed to be in parallel with some research results [22–24]. Contrary to the results of the current study, Alhoish, 2018 concluded that academic procrastination was low among nursing students [25].

The current study highlights notable trends in coping behaviors among the studied students. Concerning maladaptive emotion regulation strategies, a significant proportion of students exhibited high levels of self-blame and rumination, with more than half and over two-fifths of the students employing these strategies. Additionally, a considerable portion frequently engaged in catastrophizing, while a smaller percentage tended to blame others as a coping mechanism for stress and anxiety. In contrast, the analysis of adaptive emotion regulation strategies reveals a different pattern. Most students demonstrated a high level of engagement in positive reappraisal and refocusing on planning, with less than two-thirds employing these strategies.

Moreover, a substantial proportion utilized acceptance as a coping strategy. This can be explained by the students’ academic achievement in the first year (20.2% had excellent & and 63% had very well), which increases their self-confidence and the ability to cope with anxiety and stress as mature adults. A high level of anxiety reflects the students’ feeling of obligation to succeed and to maintain, at least, their academic achievement. Adoption of positive reappraisal and refocusing on planning is supported by the theory that students in difficult situations often rely on problem-focused rather than emotion-focused strategies and adopt adaptive coping strategies [9–11].

In nursing education, anxiety can benefit nursing students, acting as a catalyst for understanding various situations. However, prolonged exposure to anxiety can hinder students’ ability to effectively employ positive coping mechanisms. While moderate levels of anxiety can enhance student performance and serve as a motivational factor for further achievements, excessive anxiety can have detrimental effects on both performance and overall well-being, leading students to resort to maladaptive coping strategies such as avoidance behaviors. In situations where students struggle to address anxiety in the short term, they may opt for maladaptive coping strategies, such as taking breaks or vacations to temporarily alleviate stressors [1, 26–28].

It is evident in the current study that procrastination significantly predicts higher levels of academic anxiety, highlighting the detrimental impact of delaying tasks on students’ psychological well-being. However, the findings also reveal a crucial distinction between adaptive and maladaptive emotion regulation strategies. On the one hand, the utilization of adaptive coping mechanisms demonstrates a significant negative correlation with academic anxiety, indicating their effectiveness in mitigating the adverse effects of procrastination. Conversely, reliance on maladaptive coping strategies exacerbates academic anxiety, exacerbating the already elevated levels associated with procrastination. The mediating effect analyses further confirm these patterns, showing that both adaptive and maladaptive emotion regulation strategies serve as mediators in the relationship between procrastination and academic anxiety. In terms of coping strategies, a study conducted during the pandemic found that adaptive coping strategies were associated with better quality of life and work, while maladaptive coping strategies were associated with increased phobia, stress, and anxiety [29].

### Limitations

The study explores the stressors faced by emergency nursing students and the coping strategies they used to deal with them. Convenience sampling, while a common non-probability method, has notable limitations. This technique can introduce selection bias, as the sample may not accurately represent the entire population, leading to skewed results. Additionally, voluntary response bias can occur, where individuals who choose to participate may have different characteristics than those who do not, affecting the study’s outcomes. Consequently, findings from a convenience sample may lack generalizability, limiting the study’s external validity. The tools used to collect the study’s data were self-reported questionnaires, which cannot exclude bias associated with self-assessment and limit the student’s ability to report their inferences in detail. A cross-sectional research design was a limitation that prevented the establishment of causality. Since data is collected at a single point, it is challenging to determine whether the exposure preceded the outcome or vice versa. This temporal ambiguity makes it difficult to draw definitive conclusions about cause-and-effect relationships. Additionally, the susceptibility to certain biases, such as selection and recall, can affect the validity of the findings. Despite these limitations, cross-sectional studies remain valuable for providing a snapshot of the prevalence and characteristics of a population at a given time.

## Conclusion and recommendations

Research indicates that procrastination correlates positively with academic anxiety, while adaptive emotion regulation strategies such as positive reassessment and planning correlate negatively, mitigating academic anxiety. Conversely, maladaptive emotion regulation strategies like self-blame exacerbate academic anxiety. Both adaptive and maladaptive emotion regulation mechanisms mediate the relationship between anxiety perceptions and academic procrastination. Further exploration across academic levels is needed for broader understanding and comparison. Longitudinal studies are recommended to observe changes in anxiety and procrastination throughout programs. Additionally, research should investigate differences in faculty and student perspectives on learning hindrances and practical strategies, with qualitative studies offering more profound insights into stressors, anxiety, and coping.

### Nursing implications

Nursing educators must become acutely aware of the pervasive and significant levels of anxiety as well as the tendencies towards academic procrastination that are frequently exhibited by nursing students within the educational environment. To address these challenges effectively, implementing educational interventions and support programs specifically designed to enhance critical skills such as time management, stress management techniques, and adaptive emotion regulation strategies is essential. Incorporating stress reduction techniques, alongside relaxation exercises, into the nursing curriculum can serve as a vital resource for students, ultimately equipping them with the necessary tools to manage their anxiety more effectively. Furthermore, nurses who are engaged in educational settings must maintain a vigilant approach in identifying those students who may be grappling with elevated levels of anxiety and tendencies towards academic procrastination, as this proactive stance can significantly impact student outcomes. Early identification of such issues is crucial, enabling timely intervention and support, thereby preventing the escalation of these challenges, which could otherwise detrimentally affect both students’ academic performance and overall well-being. It is, therefore, of utmost importance that nursing educators strongly emphasize adopting adaptive coping strategies, which may include techniques such as positive reappraisal, systematic refocusing on planning, and the practice of acceptance in the face of stressors.

Additionally, imparting practical problem-solving skills to students, coupled with encouraging them to seek support from their peers, faculty members, and counseling services, can significantly contribute to developing effective adaptive coping mechanisms. Through such comprehensive educational efforts, nursing educators can play a pivotal role in fostering an environment that promotes resilience and success among nursing students. Their influence is significant, as they can guide students toward effective coping strategies and support systems. Ultimately, this holistic approach to education addresses immediate concerns related to anxiety and procrastination and equips students with lifelong skills that will serve them well throughout their professional careers. In conclusion, integrating these strategies into nursing education is not merely beneficial; it is imperative for cultivating competent and emotionally resilient healthcare professionals.

## Data Availability

Data will be available from the authors on reasonable request.
